# Structure-Based Virtual Screening for Drug Discovery: a Problem-Centric Review

**DOI:** 10.1208/s12248-012-9322-0

**Published:** 2012-01-27

**Authors:** Tiejun Cheng, Qingliang Li, Zhigang Zhou, Yanli Wang, Stephen H. Bryant

**Affiliations:** National Center for Biotechnology Information, National Library of Medicine, National Institutes of Health, 8600 Rockville Pike, Bethesda, Maryland 20894 USA

**Keywords:** docking, machine learning, structure-based virtual scoring, target-biased scoring function

## Abstract

Structure-based virtual screening (SBVS) has been widely applied in early-stage drug discovery. From a problem-centric perspective, we reviewed the recent advances and applications in SBVS with a special focus on docking-based virtual screening. We emphasized the researchers’ practical efforts in real projects by understanding the ligand-target binding interactions as a premise. We also highlighted the recent progress in developing target-biased scoring functions by optimizing current generic scoring functions toward certain target classes, as well as in developing novel ones by means of machine learning techniques.

## INTRODUCTION

The discovery of innovative leads with potential interaction to specific targets is of central importance to the early-stage drug discovery. This is conventionally achieved by wet-lab high-throughput screening (HTS), an established technology adopted by pharmaceutical industry. On the other hand, the high cost and low hit rate associated with HTS have stimulated the development of computational alternatives and the broad application of the cheaper and faster screening *in silico* (1,2). The completion of the Human Genome Project has revealed a wealth of attractive druggable targets (3). Meanwhile, structure biology advances in X-ray crystallography and nuclear magnetic resonance spectroscopy have further opened doors to structure-based virtual screening (SBVS) by offering in-depth structural details of these targets as well as their interactions with ligands (4,5).

There have been a mounting number of success stories reported by use of SBVS (4,6), among which docking-based virtual screening (DBVS) is arguably the most widely applied one in practice (7). Here, we reviewed the recent advances and applications in SBVS from a problem-centric perspective with a focus on DBVS, such as the practical aspects about enriching screening library before docking, considering target flexibility, metal ions, water molecules, and other key ligand–target interactions and environmental factors during docking and improving pose/compound selection after docking. We emphasized the importance of profound knowledge of the targets and/or their interactions with ligands to a successful project. We also highlighted the recent progress in developing target-biased scoring function and the trend in applying machine learning techniques to build scoring functions. As the area of DBVS is often actively reviewed, we confined our survey to the primary publications since 2007 within a 5-year time frame.

## DOCKING-BASED VIRTUAL SCREENING

**Fig. 1:**
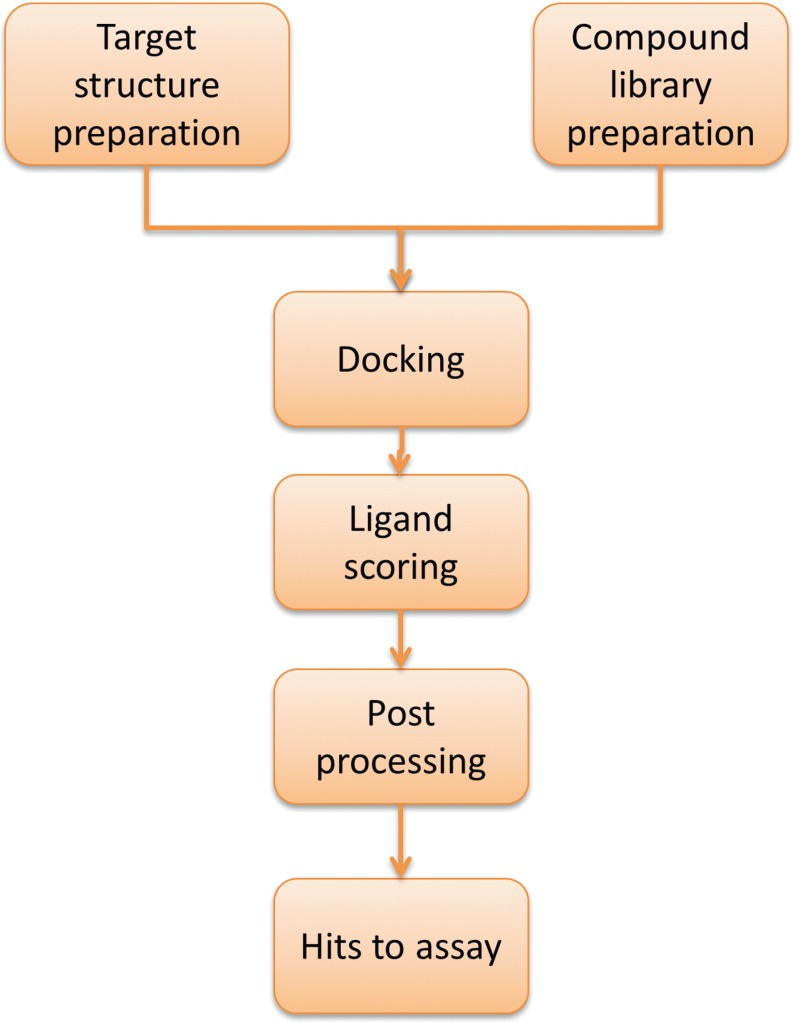
Typical workflow of a docking-based virtual screening (DBVS)

The basic inputs of a typical DBVS workflow are a target structure, either experimentally solved or computationally modeled, and a compound library of small molecules available via purchase or synthesis (Fig. 1). Often, both the target and the compound library require preparations, such as assigning proper tautomeric, stereoisomeric, and protonation states (8,9). Each compound in the library is virtually docked into the target binding site through a docking program, which computationally models the ligand–target interaction to achieve an optimal complementarity of steric and physicochemical properties. A mathematical algorithm (referred to as “scoring function”) is then used to evaluate the fitness between the docked compound and the target. This is often followed by a post-processing step, in which compounds were ranked and selected on the basis of calculated binding scores and/or other criteria, and usually only a small group of top-ranked compounds will be chosen as candidates for later experimental assays. During the past decades, a large number of docking programs have been developed (10–18). Among the most popular ones are AutoDock, Dock, FlexX, Glide, Gold, Surflex, ICM, LigandFit, and eHiTS, to name only a few (Table I).

**Table I:** Examples of Widely Used Docking Programs

Program	Search strategy	Free for academia	Website
AutoDock (10)	GA/MC	Yes	http://autodock.scripps.edu
Dock (11)	IC	Yes	http://dock.compbio.ucsf.edu
FlexX (12)	IC	No	http://www.biosolveit.de/flexx
Glide (13)	Hybrid	No	http://www.schrodinger.com
Gold (14)	GA	No	http://www.ccdc.cam.ac.uk/products/life_sciences/gold
Surflex (15)	IC	No	http://www.tripos.com/index.php
ICM (16)	MC	No	http://www.molsoft.com/docking.html
LigandFit (17)	MC	No	http://accelrys.com/products/discovery-studio
eHiTS (18)	IC	No	http://www.simbiosys.ca/ehits/index.html

*GA* genetic algorithm, *MC* Monte Carlo, *IC* incremental construction

Substantial process in DBVS requires a deep knowledge of the nature of the designated target system and/or the ligand–target binding mechanism (6). It thus seems more appropriate in many applications to view DBVS from a problem-centric than a method-centric perspective (19). In this work, we provided a review by focusing on the knowledge-based practices and efforts that were adopted by researchers throughout the workflow of DBVS (Fig. 1). General advances in the ligand conformational sampling algorithms of docking programs have been extensively reviewed elsewhere (7,20–24) and were thus not covered here.

### Enriching Compound Library before Docking

It is well accepted that the content and quality of a compound library have pivotal effects on the success of a DBVS project (25). Table II summarizes an incomplete list of public and commercial chemical databases that are commonly screened in real practices. These databases often contain a vast amount of small-molecule compounds varying from several tens of thousands to several millions. Despite the increasing power of modern computers, a blind docking with all library compounds often leads to a waste of time and computer resource. Moreover, it will impose a great burden on later compound selection. Therefore, it would be always wise to remove undesirable compounds and select only relevant ones from a library before the cost-intensive docking. A common strategy is to apply fast physicochemical filters inspired by the rule of five (26) or ligand-based similarity search seeded with known active ligands (27).

**Table II:** Commonly Screened Chemical Databases

Database	Type	No. of compounds^*a*^	Website
PubChem	Public	30 million	http://pubchem.ncbi.nlm.nih.gov
ChEMBL	Public	1 million	https://www.ebi.ac.uk/chembldb/index.php
NCI Set	Public	140,000	http://dtp.nci.nih.gov/index.html
ChemSpider	Public	26 million	http://www.chemspider.com
CoCoCo	Public	7 million	http://cococo.unimore.it/tiki-index.php
TCM	Public	32,000	http://tcm.cmu.edu.tw
ZINC	Public	13 million	http://zinc.docking.org
ChemBridge	Commercial	700,000	http://www.chembridge.com
Specs	Commercial	240,000	http://www.specs.net
Asinex	Commercial	550,000	http://www.asinex.com
Enamine	Commercial	1.7 million	http://www.enamine.net
Maybridge	Commercial	56,000	http://www.maybridge.com
WOMBAT	Commercial	263,000	http://www.sunsetmolecular.com
ChemDiv	Commercial	1.5 million	http://www.chemdiv.com
ChemNavigator	Commercial	55.3 million	http://www.chemnavigator.com
ACD	Commercial	3,870,000	http://accelrys.com/products/databases/sourcing/available-chemicals-directory.html
MDDR	Commercial	150,000	http://accelrys.com/products/databases/bioactivity/mddr.html

^*a*^Approximate numbers

A more object-oriented and efficient approach might be designing a focused library for specific targets. For example, Gozalbes *et al*. have enriched a kinase-targeted compound library using kinase-specific filters, which were derived from systematic docking and scoring of 123 diverse ligands against three kinases with known crystal structures (28). For each kinase, the filter is constructed in two steps. First, the highest score given by a certain scoring function among all docking poses of a known ligand is used as the score for this ligand. Second, the lowest score among all known ligands is selected as the threshold for the current scoring function. Combining all thresholds from six scoring functions comprises the final filter. This method was validated by testing 60 compounds, which were split evenly into two groups including those passed all the thresholds and the rest. An overall 6.7-fold higher hit rate was obtained for the first group. Likewise, Sage *et al*. (29) have introduced the GA-focused descriptor active space (GAFDAS) method to design a focused chemical space for G-protein coupled receptors by selecting target-specific descriptors through genetic algorithm. Though their method was validated in the context of ligand-based virtual screening, it could be applied in SBVS to design enriched library as well.

Structural details from observed ligand–target complexes are useful to derive pharmacophoric filters, which may be used for enriching a library with compounds that satisfy specific geometric and/or physicochemical constraints. For instance, Kireev *et al*. (30) have applied the Discovery Studio software to construct a pharmacophore model including a hydrogen bond donor (HBD), a hydrogen bond acceptor (HBA), and an amine cation involved in an ionic bond with the Asp355 residue that are observed in the crystal structure of L3MBTL1 protein in complex with H4K20me2 ligand. With these pharmacophoric constraints, the original 5,888,263 compounds were dramatically reduced to 20,078 compounds, which were subsequently subject to docking analysis. Similarly, Lee *et al*. have constructed two pharmacophore models for vascular endothelial growth factor kinase 2 (VEGFR2) using a crystal complex structure and validated them with 15 known VEGFR2 inhibitors (31). In their study, a set of 59,600 compounds was narrowed down to 16,000 and 19,100 compounds using the above two pharmacophore models as queries, respectively. In the absence of experimental structure of target, a homology model can also be indicative for analyzing the key ligand–target interactions. For example, in an attempt to discover novel inhibitors of protein arginine methyltransferase 1 (PRMT1), Heinke *et al*. have defined a structure-based pharmacophore model based on a homology structure of PRMT1 in complex with *S*-adenosylhomocysteine (32). The 6,232 compounds that matched the pharmacophoric features (one HBD, one HBA, and two hydrophobic/aromatic constraints) were enriched from the initial 189,000 compounds for subsequent docking study.

### Understanding Ligand–Target Interaction and Environmental Factors During Docking

#### Target Flexibility

Molecular targets are dynamic in their physiological environment, which are often crucial for various biological functions. The target binding pocket often adapts upon ligand binding to fit the ligands through various conformational changes ranging from small side-chain flip to large loop shift. Nevertheless, the experimentally solved target structures or ligand–target complex structures are basically static snapshots. Though previous works have shown that proper consideration of target flexibility can improve DBVS results (33), it still represents one of the greatest challenges for current docking programs (34) and becomes a hot issue in recent DBVS studies (35–39).

Ensemble docking that takes advantage of multiple target conformers has emerged as a partial solution to account for target flexibility in docking. The MultiCopyMD method developed by Okamoto *et al*. can generate a target ensemble through molecular dynamics (MD) with multiple ligands in the target binding site simultaneously (40). Applying this target ensemble in their SBVS for novel inhibitors of death-associated protein kinase (DAPK), they discovered a highly potent (IC_50_ = 69 nM) and selective inhibitor for DAPK1. To select appropriate target conformers, Rueda *et al*. have suggested a simple recipe by choosing the target conformers co-crystallized with the largest ligands (41), providing higher selectivity and better results than randomly picked ones when combined in ensemble. Using cyclin-dependent kinase 2 (CDK2) as a test example, Sperandio *et al*. have demonstrated normal mode analysis as an effective tool to select relevant target conformations with diverse binding sites (42). Generally in ensemble docking, an individual docking run is required for each target conformation, which is thus computationally inefficient. To address this issue, Bottegoni *et al*. have proposed a 4D docking approach that allows fast and accurate account of target conformational ensembles in a single docking simulation (43). This is achieved by merging 3D grids from optimally superimposed multiple target conformers into a single 4D object.

#### Metal Ions

Some targets, such as metalloproteins, contain transition metal ions in their binding sites. The binding of ligand to these targets can be substantially distinct from other target types since such metal ions often coordinate ligand polar atoms, which may help to place and orient the ligand correctly in the binding sites. However, it is nontrivial to take metal ions into account accurately in current docking/scoring algorithms. The neglection of them would inevitably lead to underestimation of the metal–ligand interaction or even incorrectly docked ligands. Therefore, increasing attentions are being paid to metal ions in recent DBVS.

Röhrig *et al*. have studied the irons in heme proteins and demonstrated their importance for DBVS (44). Two docking runs were performed in parallel by using a test set of 50 heme-containing complexes with iron–ligand contact. In one standard docking using EADock, a success rate of only 28% was achieved, clearly indicating the underestimation of the role of iron–ligand interactions. They then introduced the Morse-like metal binding potentials into EADock, which were fitted to reproduce density functional theory calculations. As a result, the success rate was doubled to 62%. To evaluate the reliability of the chosen docking protocol for screening potent cytochrome P450 aromatase inhibitors (AIs), Caporuscio *et al*. investigated a set of known imidazole and triazole AIs and found that the Glide docking program failed to predict a correct binding mode in all cases where the azole nitrogen coordinates the heme iron (45). This observation inspired them to set up a metal constraint in Glide, which requires that a ligand atom lies within a certain region of the binding site in order to interact with specific target functionalities. Their structure-based design efforts eventually resulted in several novel AIs with IC_50_ activity in the range of 21.7 μM to 9.4 nM.

Missing parameters of zinc ions is another common barrier for docking many metalloenzymes including histone deacetylases (HDACs). In seek of novel HDAC inhibitors, Park *et al*. derived potential parameters for zinc ions following a standard procedure (46), in which geometry optimization of a simplified structural model was conducted for the active-site zinc ion cluster in complex with a hydroxamate-based inhibitor at the B3LYP/6–31 G** theory level. With these zinc parameters, they discovered six novel HDAC inhibitors with IC_50_ value ranging from 1 to 100 μM.

#### Water Molecules

There is a recognition that active-site water molecules play an important role in ligand-target binding (47). Such water molecules can significantly contribute enthalpically and entropically to ligand–target binding. The most known role of water molecules is to mediate the ligand–target interaction by forming hydrogen bonds at the interface between the ligand and the target. On the other hand, the presence (or absence) and the location of water molecules may vary largely among ligands (48). Despite their critical role, accounting for water molecules accurately in docking is a long-standing challenge. Several very recent studies directly targeted this issue.

Abel and coworkers have developed a unique approach WaterMap (49) to account for the contribution of the displacement of water molecules by ligand to binding free energy. It first identifies “hydration sites” in the active site by clustering the trajectories from MD simulation of a solvated target with explicit water molecules. Inhomogeneous solvation theory is then applied to compute the thermodynamic properties of these active-site solvents including enthalpic and entropic changes. A displaced solvent functional is derived to estimate the relative binding free energies of a series of congeneric ligands based on their measured free energies by displacing active-site water molecules. This feature has made WaterMap particularly suitable for (and thus also limited to) lead optimization by providing insightful guidance to medicinal chemistry. More recently, WaterMap has been augmented by the introduction of an additional term attributable to the occupation of the dry regions in the target active site by ligand atoms (50).

Lie *et al*. have proposed a very interesting approach that attached water molecules to ligand during docking (51). In their method, ligand polar atoms are solvated with maximum number of water molecules, which are then retained or displaced depending on energy contributions during docking simulation. The novelty of their method is that each water molecule is treated as a flexible on/off part of the ligand, instead of being a static part of the target. In such a manner, water molecules are sampled with the same flexibility as the ligand itself. Their method has been evaluated with considerable improvement by using 12 structurally diverse complexes, where several water molecules bridge the ligand and the target.

Rossato *et al*. have introduced a directional approach, AcquaAlta, to consider the solvation of ligand–target complexes (52). Through an extensive analysis of the Cambridge Structural Database, they derived a geometric criteria defining interactions of water molecules with ligand and target. They also evaluated the propensity of ligand hydration through *ab initio* calculations. AcquaAlta has been validated with 20 crystal structures and reproduced 76% of the positions of water molecules that were experimentally observed.

#### Other Key Interactions

Understanding of the interactions essential for ligand–target binding is critical to the success of lead discovery and optimization. For example, in a recent attempt to identify novel inhibitors of trihydroxynaphthalene reductase (3HNR) (53), the authors first overlaid the known 3HNR inhibitors and then constructed a pharmacophore model that consists of several key interaction points within the active site: H-bonds with Ser149, Tyr163, Met200, and Tyr201 and π-stacking with Tyr208. In accordance to these interactions, the docking experiment was conducted in such a way that it only considered docking solutions that predicted π-stacking with Tyr208 and an optional H-bond with Ser149. The most potent hit compound they found exhibited a *K*_*i*_ of 5.3 ± 0.3 μM against 3HNR.

As revealed by the crystal structures of kinases in complex with ATP-competing inhibitors, such inhibitors typically form at least one hydrogen bond with backbone amide or carbonyl groups in the hinge region. Therefore, introducing relevant constraints with the hinge region for the molecules docked into the ATP sites of kinases would improve the chance of finding active compounds. This has been practiced by Ravindranathan *et al*. in the hit discovery of fibroblast growth factor receptor 1 (FGFR1) (54). Among the 23 purchasable compounds suggested by a virtual screening experiment against 2.2 million compounds, two were identified to inhibit FGFR1 kinase with medium potency (IC_50_ = 23 and 50 μM, respectively).

For certain target or ligand system, specifically designed methods may be more efficient. For example, Lang *et al*. recently have optimized DOCK 6 for docking small molecules to RNA targets (55) and obtained a success rate of 70% for the ligands with less than seven rotatable bonds at the 2-Å heavy-atom root-mean-squared deviation threshold. The BALLDock/SLICK developed by Kerzmann is a ligand-specific docking approach for docking carbohydrate or carbohydrate-like compounds, which are often problematic for standard docking programs (56).

### Improving Pose/Compound Selection After Docking

Due to the poor performance of current scoring functions in estimating binding affinity and hence in ranking docked ligands, it is recognized that compound selection based on calculated scores is not sufficient and visual inspection is often necessary. However, a practical concern arises if one needs to manually inspect thousands of docking poses. Therefore, huge efforts have been devoted to automating this procedure based on the indications gained from ligand–target interactions (57).

The molecular interaction fingerprints (IFPs), which are simple bit strings that encode 3D information about ligand–target interactions into 1D binary vector, have been extended by Marcou and Rognan as a post-docking filter to prioritize the most relevant poses of low molecular weight fragments (58). In their study, IFPs were evaluated with four popular docking tools (FlexX, Glide, Gold, and Surflex) for extracting the scaffolds of true CDK2 inhibitors. They observed that scoring by the Tanimoto similarity of IFPs to a given reference was statistically superior to conventional scoring functions in placing the low molecular weight fragment in the CDK2 binding site.

Based on the assumption that active compounds should have specific contacts with their target to display activity and also to tackle the inefficiency of traditional clustering of docking poses, Bouvier *et al*. have proposed the Automatic analysis of Poses using Self-Organizing Map (AuPosSOM) method for pose ranking with careful analysis of interatomic contacts between the docked ligand and the target (59). They have demonstrated that it is possible to differentiate active compounds from inactive ones using only mean protein contacts’ footprints calculated from the multiple conformations given by docking software.

Protein-specific structural filtration has been introduced by Novikov *et al*. to improve the performance of DBVS (60). The filter was defined by a set of crucial ligand–target interactions that are structurally conserved in the available ligand-bound target structures. The application of this method achieved a substantial improvement of enrichment factor ranging from several folds to several hundreds folds against a set of ten diverse protein targets. The authors demonstrated that the structural filtration had effectively repaired the deficiencies of scoring functions, resulting in a considerably lower false positive rate.

Wei *et al*. have demonstrated that binding energy landscape analysis could help to discriminate true hits from high-scoring decoys in virtual screening (61). In their work, two parameters (*i.e.*, the energy gap and the number of local binding wells in the landscape) were used to account for the kinetic accessibility. With a linear combination of the two parameters, they obtained, in a five-fold cross-validation, the areas under the receiver operator characteristic curves (AUC) of 0.878 for neuraminidase and 0.776 for cyclooxygenase 2 (COX2), respectively. In a more independent test using the directory of useful decoys (DUD) set, the enrichment ratio given by these two parameters when combined with docking scores was improved to 200–300% as compared to that using scoring function alone.

## SCORING FUNCTIONS

Scoring function is at the heart of molecular docking by assisting a docking program to efficiently explore the binding space of a ligand. It is also responsible for evaluating the binding affinity once the correct binding pose is identified. Therefore, the predictability of scoring functions has a significant impact on the productivity of DBVS.

A multitude of scoring functions have been reported in the past decades (10–15,62–71) (Table III), and new ones are still emerging. Current scoring functions, as reviewed in other works (23,72), can be roughly classified into three types: (a) Force field-based scoring functions employ classic force field to compute the noncovalent ligand–target interactions, such as van der Waals and electrostatic energies. They are often augmented by a GB/SA or PB/SA term in order to account for solvation effects. (b) Empirical scoring functions calculate the overall binding free energy from several energetic terms, including hydrogen bond interaction and hydrophobic interaction. The weighting factors of all terms are calibrated from a set of known complexes with experimentally determined structures and binding affinities. (c) Knowledge-based scoring functions compute the ligand–target interactions as a sum of distance-dependent statistical potentials between the ligand and the target. It is notable that the deduction of such potentials needs only the structural information of ligand–target complexes, which is being accumulated rapidly due to structural biology advances.

**Table III:** Examples of Current Scoring Functions

Type	Scoring function
Force field	AutoDock (10), DOCK (11), GoldScore (14), D-Score (11)
Empirical	LUDI (62), X-Score (63), PLP (64), ChemScore (65), FlexX/F-Score (12), GlideScore (13), Surflex (15), QXP/Flo+ (66)
Knowledge based	PMF04 (67), kinase-PMF (68), DrugScore (69), ASP (70), ITScore (71)

The performance of various scoring functions has been investigated by several comparative studies (73–77), with respect to the ability of reproducing known binding pose, predicting binding affinity and rank-ordering a compound library. The state-of-the-art scoring functions are at different levels of accuracy, and it is clear that no single scoring function consistently outperforms others in all cases. It is concluded from previous comparative studies that today’s scoring functions are often capable of identifying the correct binding pose of a ligand, while binding affinity prediction with high accuracy is still far from reach (73). Therefore, considerable efforts have been made to improve the performance of current scoring functions. Common strategies include adding additional factors to account for solvation and entropic effects (71), deriving more accurate energy terms by high-level quantum calculations (78), and consensus scoring by combination of multiple scoring functions (79,80). In this review, we highlighted the recent progress in developing target-biased scoring functions as well as those employed machine learning techniques.

### Target-Biased Scoring Functions

Most of the today’s scoring functions are generic models derived from the large-scale experimental data of ligand–target complexes and are presumably applicable to all sorts of target classes. However, previous comparative studies have revealed that a universally accurate scoring function is still out of reach. A practical remedy to this might be developing target-biased alternatives for specific targets or tasks (81).

#### Target-Biased Scoring Functions Derived by Re-parameterization

The most straightforward way to obtain a target-biased scoring function is, probably, to re-calibrate an existing all-purpose scoring function directly on certain target classes. For example, DrugScore-RNA (82) adopts the same framework as DrugScore (69) but is derived from 670 crystal structures of nucleic acid–ligand and nucleic acid–protein complexes. Similar idea has been implemented in the kinase family-specific potential of mean force (kinase-PMF) (68), a kinase-targeted scoring function adjusted from the original PMF04 (67).

Tweaking the parameters in original scoring functions toward specific targets is also a prevalent strategy to derive target-biased scoring functions. For example, Teramoto and Fukunishi have applied a supervised scoring model to tailor the FlexX scoring function (*F*-score), which outperformed its former version on three of the five tested targets (83). The TOP approach suggested by Seifert (84) have employed iterative taboo search to optimize the scoring function in ProPose and the original Böhm scoring function against three targets, including CDK2, estrogen receptor, and COX2. By adding negative data of ligands that are known not to bind particular target, Pham and Jain have tuned the scoring function in Surflex-Dock and observed substantially enhanced screening enrichment for HIV protease and poly(ADP-ribose) polymerase (85). An augmented Flo+ scoring function has been developed by Catana and Stouten using N-way partial least squares (PLS) (86), which significantly improved the correlation between observed and calculated p*K*_*i*_ values from *R* ^2^ = 0.5 to 0.8 on a relatively diverse set of ligand–target complexes spanning seven protein families. Therefore, it would be attractive if scoring functions offer extendable or customizable features.

#### Target-Biased Scoring Functions Require no Re-parameterization

The above-mentioned target-biased scoring functions typically require re-parameterization or special treatment of established scoring functions. Too often, existing scoring functions are available to end-users as black boxes, hence it is not readily possible to adjust their parameters by any optimization algorithm. Several approaches have been proposed to address this issue. One of the earliest examples is the MultiScore that employs the raw scores from eight scoring functions to characterize the observed p*K*_*i*_ (87), which has been found to work better for matrix metalloproteinases. The implied idea is slightly different from that of consensus scoring (79,80) in that it assumes uneven contributions from individual scoring functions. In a similar way, the AutoShim method has incorporated the original Flo+ score as well as additional target-specific pharmacophore points (shims) as descriptors in PLS analysis (88). More recently, Cheng *et al*. have proposed a knowledge-guided strategy (KGS) based on the similarity principle aiming to improve the accuracy of binding affinity prediction of current scoring functions (89). The KGS strategy computes the binding affinity of a query ligand–target complex based on the known binding affinity of an appropriate reference complex, which is required to share a similar pattern of key ligand–target interactions to that of the query complex of interest. The KGS strategy has been validated with both observed and docked ligand–target complex structures. Moreover, it can in principle work in concert with any scoring method, and its application is not limited to specific classes of ligand–target complexes.

### Machine Learning and Scoring Functions

Machine learning techniques are powerful to construct and optimize predictive models. In recent years, there is an increasing interest in developing novel scoring functions by means of machine learning (90). A notable feature is that they take into account the commonly observed ligand–target binding interactions in an implicit manner, which obviates the need of explicitly modeling the error-prone interactions, including solvation and entropic effects. Moreover, machine learning techniques such as neural networks (NN), support vector machines (SVM), and random forest (RF) are able to account for the nonlinear dependence among the various interactions involved in ligand–target binding. As a result, despite being less concrete on the physicochemical basis, they often demonstrated a superior or at least comparable performance to that of classic scoring functions in binding affinity estimation.

The NNScore scoring function developed by Durrant and McCammon is based on NN (91), which attempts to computationally simulate the microscopic organization of human brain. The input layer consists of 194 neurodes that are related to ligand–target interactions. Kinnings *et al*. (92) have applied SVM to train a new scoring function for identifying inhibitors of *Mycobacterium tuberculosis* InhA, using the individual energy terms as descriptors obtained directly from the built-in scoring function of eHiTS. Amini *et al*. have introduced the support vector inductive logic programming as a general approach to develop system-specific scoring functions (93). The descriptors they used are the distances from each fragment’s central ligand atom to target atoms. In the development of PHOENIX scoring function, Tang *et al*. have adopted an indirect idea (94). They first modeled independently enthalpy (Δ*H*) and the change of entropy (*T*Δ*S*) by fitting relevant descriptors to experimentally measured calorimetric data through PLS and then calculated the binding free energy (Δ*G*) according to thermodynamic cycle.

Similar to the idea of using occurrence count of ligand–target atom pair as geometric descriptor to generate a scoring function (95), Li *et al*. (96) have developed a target-specific scoring method, SVM-SP, by using SVM. SVM-SP employs 135 atom pair potentials as descriptors that are derived in the same way as traditional knowledge-based scoring functions. The effectiveness of SVM-SP has been strongly supported by the discovery of three novel micromolar hits against epidermal growth factor receptor. The recently released RF-score by Ballester and Mitchell (97) has been built with RF, where a set of descriptors are introduced based on the count of a particular ligand–target atom pair within a certain distance range. Despite the relatively coarse definition of ligand–target atom pairs, which considers only atomic number with no concern about distance dependence, RF-score strikingly outperformed all 16 state-of-the-art scoring functions in a recent benchmark (73).

## SUMMARY

SBVS becomes routine in both pharmaceutical companies and academic groups for early-stage drug discovery. In this work, we reviewed the recent advances and applications in DBVS from a problem-centric perspective with an emphasis on the integration of available knowledge adopted by researchers in real practice. It is found that enriching a screening library for a specific target before docking can improve both computational efficiency and hit rate. Also, effective consideration of key ligand-target interactions and other environmental factors during docking, such as target flexibility, metal ions, and water molecules, can give enhanced DBVS performance. In addition, post-docking processing techniques that automate the selection of appropriate poses/compounds not only greatly alleviate the human intervention of docking outputs but also improve the final outcome simultaneously. Developing target-biased scoring functions represents a trend in tweaking current all-purpose alternatives toward specific target classes. Recent development of scoring function also observed an increasing use of machine learning techniques, which have an intrinsic non-linear feature and can implicitly account for some really challenging ligand–target interactions such as solvation and entropic effects.

Despite the listed advances here, current improvements in DBVS over state-of-the-art, in large part, only serve as patches or temporary remedies to existing methods, which often rely on expertise knowledge and thus may have limited applications in real practice. A universally accurate and reliable solution is still far from reach in the near future. Revolutionary innovations are definitely in urgent need and thus highly encouraged to address the fundamental challenges such as target flexibility and water molecules.

## References

[1] Ripphausen P, Nisius B, Peltason L, Bajorath Jr (2010). Quo vadis, virtual screening? A comprehensive survey of prospective applications. J Med Chem.

[2] Clark DE (2008). What has virtual screening ever done for drug discovery?. Expert Opin Drug Discov.

[3] Hopkins AL, Groom CR (2002). The druggable genome. Nat Rev Drug Discov.

[4] Villoutreix BO, Eudes R, Miteva MA (2009). Structure-based virtual ligand screening: recent success stories. Comb Chem High Throughput Screen.

[5] Ghosh S, Nie A, An J, Huang Z (2006). Structure-based virtual screening of chemical libraries for drug discovery. Curr Opin Chem Biol.

[6] Seifert MHJ, Lang M (2007). Essential factors for successful virtual screening. Mini Rev Med Chem.

[7] Tuccinardi T (2009). Docking-based virtual screening: recent developments. Comb Chem High Throughput Screen.

[8] Rapp CS, Schonbrun C, Jacobson MP, Kalyanaraman C, Huang N (2009). Automated site preparation in physics-based rescoring of receptor ligand complexes. Proteins: Struct, Funct, Bioinf.

[9] ten Brink T, Exner T (2010). pKa based protonation states and microspecies for protein–ligand docking. J Comput Aided Mol Des.

[10] Morris GM, Goodsell DS, Halliday RS, Huey R, Hart WE, Belew RK (1998). Automated docking using a Lamarckian genetic algorithm and an empirical binding free energy function. J Comput Chem.

[11] Ewing TJ, Makino S, Skillman AG, Kuntz ID (2001). DOCK 4.0: search strategies for automated molecular docking of flexible molecule databases. J Comput Aided Mol Des.

[12] Rarey M, Kramer B, Lengauer T, Klebe G (1996). A fast flexible docking method using an incremental construction algorithm. J Mol Biol.

[13] Friesner RA, Banks JL, Murphy RB, Halgren TA, Klicic JJ, Mainz DT (2004). Glide: a new approach for rapid, accurate docking and scoring. 1. Method and assessment of docking accuracy. J Med Chem.

[14] Jones G, Willett P, Glen RC, Leach AR, Taylor R (1997). Development and validation of a genetic algorithm for flexible docking. J Mol Biol.

[15] Jain AN (2003). Surflex: fully automatic flexible molecular docking using a molecular similarity-based search engine. J Med Chem.

[16] Abagyan R, Totrov M, Kuznetsov D (1994). ICM: a new method for protein modeling and design: applications to docking and structure prediction from the distorted native conformation. J Comput Chem.

[17] Venkatachalam CM, Jiang X, Oldfield T, Waldman M (2003). LigandFit: a novel method for the shape-directed rapid docking of ligands to protein active sites. J Mol Graph Model.

[18] Zsoldos Z, Szabo I, Szabo Z, Peter Johnson A (2003). Software tools for structure based rational drug design. J Mol Struct (THEOCHEM).

[19] Schneider G (2010). Virtual screening: an endless staircase?. Nat Rev Drug Discov.

[20] Pujadas G, Vaque M, Ardevol A, Blade C, Salvado MJ, Blay M (2008). Protein–ligand docking: a review of recent advances and future perspectives. Curr Pharmaceut Anal.

[21] Meng X-Y, Zhang H-X, Mezei M, Cui M (2011). Molecular docking: a powerful approach for structure-based drug discovery. Curr Comput-Aided Drug Des.

[22] Yuriev E, Agostino M, Ramsland Pa (2010). Challenges and advances in computational docking: 2009 in review. J Mol Recognit.

[23] Moitessier N, Englebienne P, Lee D, Lawandi J, Corbeil CR (2008). Towards the development of universal, fast and highly accurate docking/scoring methods: a long way to go. Br J Pharmacol.

[24] Dias R, de Azevedo Jr WF (2008). Molecular docking algorithms. Curr Drug Targets.

[25] Cummings MD, Maxwell AC, DesJarlais RL (2007). Processing of small molecule databases for automated docking. Med Chem.

[26] Lipinski CA, Lombardo F, Dominy BW, Feeney PJ (1997). Experimental and computational approaches to estimate solubility and permeability in drug discovery and development settings. Adv Drug Deliv Rev.

[27] Perez-Pineiro R, Burgos A, Jones DC, Andrew LC, Rodriguez H, Suarez M (2009). Development of a novel virtual screening cascade protocol to identify potential trypanothione reductase inhibitors. J Med Chem.

[28] Gozalbes R, Simon L, Froloff N, Sartori E, Monteils C, Baudelle R (2008). Development and experimental validation of a docking strategy for the generation of kinase-targeted libraries. J Med Chem.

[29] Sage C, Wang R, Jones G. G-protein coupled receptors virtual screening using genetic algorithm focused chemical space. J Chem Inf Model. 2011. doi:10.1021/ci200043z. 10.1021/ci200043z 21761904

[30] Kireev D, Wigle TJ, Norris-Drouin J, Herold JM, Janzen WP, Frye SV (2010). Identification of non-peptide malignant brain tumor (MBT) repeat antagonists by virtual screening of commercially available compounds. J Med Chem.

[31] Lee K, Jeong K-W, Lee Y, Song JY, Kim MS, Lee GS (2010). Pharmacophore modeling and virtual screening studies for new VEGFR-2 kinase inhibitors. Eur J Med Chem.

[32] Heinke R, Spannhoff A, Meier R, Trojer P, Bauer I, Jung M (2009). Virtual screening and biological characterization of novel histone arginine methyltransferase PRMT1 inhibitors. Chem Med Chem.

[33] Rueda M, Bottegoni G, Abagyan R (2009). Consistent improvement of cross-docking results using binding site ensembles generated with elastic network normal modes. J Chem Inf Model.

[34] Cavasotto CN, Singh N (2008). Docking and high throughput docking: successes and the challenge of protein flexibility. Curr Comput-Aided Drug Des.

[35] Cozzini P, Kellogg GE, Spyrakis F, Abraham DJ, Costantino G, Emerson A (2008). Target flexibility: an emerging consideration in drug discovery and design. J Med Chem.

[36] Durrant JD, McCammon JA (2010). Computer-aided drug-discovery techniques that account for receptor flexibility. Curr Opin Pharmacol.

[37] Sotriffer CA (2011). Accounting for induced-fit effects in docking: what is possible and what is not?. Curr Top Med Chem.

[38] Lill MA (2011). Efficient incorporation of protein flexibility and dynamics into molecular docking simulations. Biochemistry.

[39] Lin J-H (2011). Accommodating protein flexibility for structure-based drug design. Curr Top Med Chem.

[40] Okamoto M, Takayama K, Shimizu T, Ishida K, Takahashi O, Furuya T (2009). Identification of death-associated protein kinases inhibitors using structure-based virtual screening. J Med Chem.

[41] Rueda M, Bottegoni G, Abagyan R (2009). Recipes for the selection of experimental protein conformations for virtual screening. J Chem Inf Model.

[42] Sperandio O, Mouawad L, Pinto E, Villoutreix B, Perahia D, Miteva M (2010). How to choose relevant multiple receptor conformations for virtual screening: a test case of Cdk2 and normal mode analysis. Eur Biophys J.

[43] Bottegoni G, Kufareva I, Totrov M, Abagyan R (2008). Four-dimensional docking: a fast and accurate account of discrete receptor flexibility in ligand docking. J Med Chem.

[44] Röhrig UF, Grosdidier A, Zoete V, Michielin O (2009). Docking to heme proteins. J Comput Chem.

[45] Caporuscio F, Rastelli G, Imbriano C, Del Rio A (2011). Structure-based design of potent aromatase inhibitors by high-throughput docking. J Med Chem.

[46] Park H, Kim S, Kim YE, Lim S-J (2010). A structure-based virtual screening approach toward the discovery of histone deacetylase inhibitors: identification of promising zinc-chelating groups. Chem Med Chem.

[47] Thilagavathi R, Mancera RL (2010). Ligand–protein cross-docking with water molecules. J Chem Inf Model.

[48] Santos R, Hritz J, Oostenbrink C (2009). Role of water in molecular docking simulations of cytochrome P450 2D6. J Chem Inf Model.

[49] Abel R, Young T, Farid R, Berne BJ, Friesner RA (2008). Role of the active-site solvent in the thermodynamics of factor Xa ligand binding. J Am Chem Soc.

[50] Wang L, Berne BJ, Friesner RA (2011). Ligand binding to protein-binding pockets with wet and dry regions. Proc Natl Acad Sci.

[51] Lie MA, Thomsen R, Pedersen CNS, Schiøtt B, Christensen MH (2011). Molecular docking with ligand attached water molecules. J Chem Inf Model.

[52] Rossato G, Ernst B, Vedani A, Smieško M. AcquaAlta: a directional approach to the solvation of ligand–protein complexes. J Chem Inf Model. 2011. doi:10.1021/ci200150p. 10.1021/ci200150p 21714532

[53] Brunskole Švegelj M, Turk S, Brus B, Lanišnik Rižner T, Stojan J, Gobec S (2011). Novel inhibitors of trihydroxynaphthalene reductase with antifungal activity identified by ligand-based and structure-based virtual screening. J Chem Inf Model.

[54] Ravindranathan KP, Mandiyan V, Ekkati AR, Bae JH, Schlessinger J, Jorgensen WL (2010). Discovery of novel fibroblast growth factor receptor 1 kinase inhibitors by structure-based virtual screening. J Med Chem.

[55] Lang PT, Brozell SR, Mukherjee S, Pettersen EF, Meng EC, Thomas V (2009). DOCK 6: combining techniques to model RNA–small molecule complexes. RNA.

[56] Kerzmann A, Fuhrmann J, Kohlbacher O, Neumann D (2008). BALLDock/SLICK: a new method for protein–carbohydrate docking. J Chem Inf Model.

[57] Waszkowycz B (2008). Towards improving compound selection in structure-based virtual screening. Drug Discov Today.

[58] Marcou G, Rognan D (2007). Optimizing fragment and scaffold docking by use of molecular interaction fingerprints. J Chem Inf Model.

[59] Bouvier G, Evrard-Todeschi N, Girault J-P, Bertho G (2010). Automatic clustering of docking poses in virtual screening process using self-organizing map. Bioinformatics.

[60] Novikov F, Stroylov V, Stroganov O, Chilov G (2010). Improving performance of docking-based virtual screening by structural filtration. J Mol Model.

[61] Wei D, Zheng H, Su N, Deng M, Lai L (2010). Binding energy landscape analysis helps to discriminate true hits from high-scoring decoys in virtual screening. J Chem Inf Model.

[62] Böhm H-J (1998). Prediction of binding constants of protein ligands: a fast method for the prioritization of hits obtained from de novo design or 3D database search programs. J Comput Aided Mol Des.

[63] Wang R, Lai L, Wang S (2002). Further development and validation of empirical scoring functions for structure-based binding affinity prediction. J Comput Aided Mol Des.

[64] Gehlhaar DK, Verkhivker GM, Rejto PA, Sherman CJ, Fogel DR, Fogel LJ (1995). Molecular recognition of the inhibitor AG-1343 by HIV-1 protease: conformationally flexible docking by evolutionary programming. Chem Biol.

[65] Eldridge MD, Murray CW, Auton TR, Paolini GV, Mee RP (1997). Empirical scoring functions: I. The development of a fast empirical scoring function to estimate the binding affinity of ligands in receptor complexes. J Comput Aided Mol Des.

[66] McMartin C, Bohacek RS (1997). QXP: powerful, rapid computer algorithms for structure-based drug design. J Comput Aided Mol Des.

[67] Muegge I (2005). PMF scoring revisited. J Med Chem.

[68] Xue M, Zheng M, Xiong B, Li Y, Jiang H, Shen J (2010). Knowledge-based scoring functions in drug design. 1. Developing a target-specific method for kinase−ligand interactions. J Chem Inf Model.

[69] Gohlke H, Hendlich M, Klebe G (2000). Knowledge-based scoring function to predict protein–ligand interactions. J Mol Biol.

[70] Mooij WTM, Verdonk ML (2005). General and targeted statistical potentials for protein–ligand interactions. Proteins: Struct, Funct, Bioinf.

[71] Huang S-Y, Zou X (2010). Inclusion of solvation and entropy in the knowledge-based scoring function for protein–ligand interactions. J Chem Inf Model.

[72] Huang S-Y, Grinter SZ, Zou X (2010). Scoring functions and their evaluation methods for protein-ligand docking: recent advances and future directions. Phys Chem Chem Phys.

[73] Cheng T, Li X, Li Y, Liu Z, Wang R (2009). Comparative assessment of scoring functions on a diverse test set. J Chem Inf Model.

[74] Warren GL, Andrews CW, Capelli A-M, Clarke B, LaLonde J, Lambert MH (2006). A critical assessment of docking programs and scoring functions. J Med Chem.

[75] Ferrara P, Gohlke H, Price DJ, Klebe G, Brooks CL (2004). Assessing scoring functions for protein–ligand interactions. J Med Chem.

[76] Wang R, Lu Y, Fang X, Wang S (2004). An extensive test of 14 scoring functions using the PDBbind refined set of 800 protein–ligand complexes. J Chem Inf Comput Sci.

[77] Wang R, Lu Y, Wang S (2003). Comparative evaluation of 11 scoring functions for molecular docking. J Med Chem.

[78] Raub S, Steffen A, Kämper A, Marian CM (2008). AIScore: chemically diverse empirical scoring function employing quantum chemical binding energies of hydrogen-bonded complexes. J Chem Inf Model.

[79] Charifson PS, Corkery JJ, Murcko MA, Walters WP (1999). Consensus scoring: a method for obtaining improved hit rates from docking databases of three-dimensional structures into proteins. J Med Chem.

[80] Wang R, Wang S (2001). How does consensus scoring work for virtual library screening? An idealized computer experiment. J Chem Inf Comput Sci.

[81] Seifert MHJ (2009). Targeted scoring functions for virtual screening. Drug Discov Today.

[82] Pfeffer P, Gohlke H (2007). DrugScoreRNA: knowledge-based scoring function to predict RNA–ligand interactions. J Chem Inf Model.

[83] Teramoto R, Fukunishi H (2007). Supervised scoring models with docked ligand conformations for structure-based virtual screening. J Chem Inf Model.

[84] Seifert MHJ (2008). Optimizing the signal-to-noise ratio of scoring functions for protein–ligand docking. J Chem Inf Model.

[85] Pham T, Jain A (2008). Customizing scoring functions for docking. J Comput Aided Mol Des.

[86] Catana C, Stouten PFW (2007). Novel, customizable scoring functions, parameterized using N-PLS, for structure-based drug discovery. J Chem Inf Model.

[87] Terp GE, Johansen BN, Christensen IT, Jørgensen FS (2001). A new concept for multidimensional selection of ligand conformations (MultiSelect) and multidimensional scoring (MultiScore) of protein–ligand binding affinities. J Med Chem.

[88] Martin EJ, Sullivan DC (2008). AutoShim: empirically corrected scoring functions for quantitative docking with a crystal structure and IC50 training data. J Chem Inf Model.

[89] Cheng T, Liu Z, Wang R (2010). A knowledge-guided strategy for improving the accuracy of scoring functions in binding affinity prediction. BMC Bioinf.

[90] Hecht D, Fogel GB (2009). Computational intelligence methods for docking scores. Curr Comput-Aided Drug Des.

[91] Durrant JD, McCammon JA (2010). NNScore: a neural-network-based scoring function for the characterization of protein–ligand complexes. J Chem Inf Model.

[92] Kinnings SL, Liu N, Tonge PJ, Jackson RM, Xie L, Bourne PE (2011). A machine learning-based method to improve docking scoring functions and its application to drug repurposing. J Chem Inf Model.

[93] Amini A, Shrimpton PJ, Muggleton SH, Sternberg MJE (2007). A general approach for developing system-specific functions to score protein–ligand docked complexes using support vector inductive logic programming. Proteins: Struct, Funct, Bioinf.

[94] Tang YT, Marshall GR (2011). PHOENIX: a scoring function for affinity prediction derived using high-resolution crystal structures and calorimetry measurements. J Chem Inf Model.

[95] Deng W, Breneman C, Embrechts MJ (2004). Predicting protein–ligand binding affinities using novel geometrical descriptors and machine-learning methods. J Chem Inf Comput Sci.

[96] Li L, Khanna M, Jo I, Wang F, Ashpole NM, Hudmon A (2011). Target-specific support vector machine scoring in structure-based virtual screening: computational validation, *in vitro* testing in kinases, and effects on lung cancer cell proliferation. J Chem Inf Model.

[97] Ballester PJ, Mitchell JBO (2010). A machine learning approach to predicting protein–ligand binding affinity with applications to molecular docking. Bioinformatics.

